# Abundance and Significance of Neuroligin-1 and Neurexin II in the Enteric Nervous System of Embryonic Rats

**DOI:** 10.1155/2017/1209360

**Published:** 2017-01-18

**Authors:** Dongming Wang, Jingnian Pan, Guoxin Song, Ni Gao, Yi Zheng, Qiangye Zhang, Aiwu Li

**Affiliations:** ^1^Department of Pediatric Surgery, Qilu Hospital, Shandong University, Shandong, China; ^2^Internal Medicine, Qingzhou Clinical School, Weifang Medical College, Weifang, China; ^3^Department of Pediatric Surgery, Weihai Municipal Hospital, Shandong, China

## Abstract

*Aim*. To investigate the abundance of neuroligin-1 and neurexin II in the enteric nervous system (ENS) of rats on different embryonic days and to explore their potential significance.* Methods*. The full-thickness colon specimens proximal to the ileocecal junction of rats on embryonic days 16, 18, and 20 and of newborns within 24 hours (E16, E18, E20, and Ep0) were studied, respectively. qRT-PCR was applied for detecting the expressions of neuroligin-1 and neurexin II on mRNA, and western blotting was employed for detecting their further expressions on the whole tissue. Finally, the histological appearance of neuroligin-1 and neurexin II*α* was elucidated using immunohistochemical staining.* Results*. qRT-PCR showed that the neuroligin-1 and neurexin II mRNA expressions of groups E16, E18, E20, and Ep0 increased gradually with the growth of embryonic rats (*P* < 0.05). Western blotting confirmed the increasing tendency. In immunohistochemical staining, proteins neuroligin-1 and neurexin II*α* positive cells concentrated mostly in the myenteric nerve plexus of the colon and their expressions depend on the embryonic time.* Conclusion*. Neuroligin-1 and neurexin II were both expressed in the ENS and have temporal correlation with the development of ENS, during which neuronal intestinal malformations (NIM) may occur due to their disruptions and consequent abnormal ENS development.

## 1. Introduction

Neurexins, a family of presynaptic neuronal cell-adhesion proteins of the central nervous system (CNS), were discovered as a functional receptor of *α*-latrotoxin triggering massive neurotransmitters release by stimulating synaptic vesicle exocytosis from presynaptic terminals [1]. They are encoded by three genes in vertebrates and there are more than 1000 isoforms due to extensive alternative splicing [2]. Neuroligins, a prototypical interaction partner of neurexins, are expressed predominantly at the postsynaptic terminal; coupling to presynaptic neurexins forms transsynaptic complexes to participate in the formation, differentiation, maturation, and plasticity of functional synapses [3–5].

HSCR, also known as congenital megacolon or intestinal aganglionosis, is the most common congenital disorder of ENS. It occurs in 1/5000 human live births and is characterized by the absence of myenteric and submucosal ganglion cells in the distal gut [6]. The patients with HSCR clinically present with delayed meconium, intractable abdominal distension in newborns, or severe constipation in young children. Since it was firstly described by Härald Hirschsprung in 1886 [7], it has become the best characterized disease of neuronal intestinal malformations (NIM). With increasing recognition of the enteric nervous system (ENS), there is also a spectrum of NIM with symptoms resembling HSCR despite the presence of ganglion cells in the distal gut [8], which have been proposed to be the concept of allied disorders of Hirschsprung's disease (ADHD). Unfortunately, the pathogenesis of NIM still remains unclear at present.

The ENS, an extensive network of neurons and glia within the wall of the gut, is the largest branch of the peripheral nervous system. Unlike the rest of the peripheral nervous system, it provides the intrinsic innervation of the bowel and regulates gastrointestinal functions independently. The ENS is vital to actively relax gastrointestinal smooth muscle, the absence or abnormality of which can result in tonic contraction of the bowel and functional obstruction as a consequence. The ENS arises from the neural crest, mostly from the vagal levels of the neuraxis. The enteric neural crest-derived cells (ENCDCs) invade the foregut and migrate along the gut wall and then proliferate and differentiate until they colonize the entire bowel and become wired together by synapses to form a functional nervous system finally [9–11]. Failure in these processes and consequent abnormal ENS development result in NIM.

Our previous studies found that neurexin and neuroligin are both expressed in the ENS and their significantly downregulated expression in HSCR may be involved in their pathogenesis. But the relationship between neurexin-neuroligin and the ENS development is still unknown. In this work, the abundance of neurexin II and neuroligin-1 in the ENS of rats at different embryonic days was investigated through quantitative real-time PCR, western blot, and immunofluorescence staining. We believe that our results would be helpful for further research on the formation of ENS and pathogenesis of NIM.

## 2. Materials and Methods

### 2.1. Animal Tissue Preparation

Our study was approved by the ethics committee of Qilu Hospital, Shandong University. Healthy Wistar rats at embryonic days 16 (E16), 18 (E18), and 20 (E20) and newborn Wistar rats within 24 hours (Ep0), provided by the animal facility of Shandong University, were used in this study. The rats were treated under the animal use guidelines of the Institutional Animal Care and Use Committee (IACUC) at Qilu Hospital and College of Medicine, Shandong University. The rats were put to death after anaesthesia via chloral hydrate. A segment of the full-thickness colon, 1.0 cm approximately, proximal to the ileocecal junction, was harvested. One-third of samples, prepared for immunohistochemistry staining, were preserved in 10% buffered formaldehyde for 24 hours, dehydrated, and embedded in paraffin. The rest of the embryonic and newborn colon samples were stored at −80°C in disinfected Eppendorf tubes for preparation of western blot and qRT-PCR assay.

### 2.2. Reagents

The detailed information of antibodies and primers is listed in Tables 1 and 2. Other reagents were also available commercially: Protein Extraction Kit (Beyotime, China), BCA Protein Concentration Determination Kit (Beyotime, China), SDS-PAGE Gel Preparation Kit (Beyotime, China), MiniBEST Universal RNA Isolation Kit (TaKaRa, Japan), PrimeScript™ RT Reagent Kit with gDNA Eraser (TaKaRa, Japan), and SYBR®* Premix Ex Taq*™ Tli RNaseH Plus (TaKaRa, Japan).

### 2.3. qRT-PCR Assay

MiniBEST Universal RNA Isolation Kit was chosen to isolate total RNA from 20 mg full-thickness colon specimens of E16, E18, E20, and Ep0. After assessing the concentration, 1 *μ*g of total RNA was used for a cDNA synthesis reaction according to the manufacturer's instructions of PrimeScript RT reagent Kit. The reaction solution was prepared according to instructions of SYBR* Premix Ex Taq* quantitative fluorescence kit and qRT-PCR reaction was performed using an instrument with Roche LightCycler® 480 system. The primers applied for qRT-PCR reaction are shown in Table 2. Then, Ct of neuroligin-1, neurexin II, and *β*-actin was measured to calculate ΔCt (Ct of neuroligin-1 or neurexin II minus Ct of *β*-actin). The Ep0 group served as a control and 2^−ΔΔCt^ was calculated finally as the relative expressions of E16, E18, and E20 for further analysis.

### 2.4. Western Blot Analysis

Total proteins were isolated from specimens of E16, E18, E20, and Ep0 according to the manufacturer's instructions of the Protein Extraction Kit. To normalize the amount of total protein, the concentrations were measured using a BCA Protein Concentration Determination Kit. Then, 50 *μ*g of total proteins was separated on 10% SDS-PAGE and transferred to PVDF membranes electrophoretically. After blocking by 5% nonfat milk for 2 hours at room temperature, membranes were incubated with primary antibodies (Table 1) at 4°C overnight and polyperoxidase-conjugated secondary antibodies (Table 1) for 1 hour at room temperature sequentially. Then, the membranes were detected by ECL system finally. Between each step, rinsing with 0.1 M TBST (pH 7.4, 5 min × 3 times) was performed. The Gel-Pro Analyzer 4.0 software was applied for calculating the abundance of neuroligin-1 and neurexin II*α*, which were expressed by the relative gray values (neuroligin-1 IOD/*β*-actin IOD, neurexin II*α* IOD/*β*-actin IOD).

### 2.5. Immunohistochemistry Staining

The paraffin-embedded blocks of E16, E18, E20, Ep0, and adult were cut into 3.0 *μ*m coronal sections. Then, the sections were dewaxed in xylene and graded alcohol. After antigen retrieval with 0.01 M citrate buffer (pH 7.4) and blocking endogenous peroxidases by 3% H_2_O_2_, the sections were blocked at nonspecific binding sites with 3% diluted goat serum for 45 min at 37°C and incubated in primary antibodies (Table 1) at 4°C overnight. Polymer helper and polyperoxidase-conjugated secondary antibodies (Table 1) were incubated at 37°C for 30 min sequentially. Then, DAB was added to stain the sections. Between each step, rinsing with 0.1 M PBST (pH 7.4, 5 min × 3 times) was performed. After dehydration with graded alcohol and clearing with xylene, the sections were coverslipped and examined finally.

### 2.6. Statistical Analyses

All data of this study is shown as mean ± SD, and *P* values < 0.05 were considered to be statistically significant. Data analyses were performed using SPSS 13.0 software. Variance test and one-way ANOVA were used for multiple comparisons of measurement data.

## 3. Results

### 3.1. qRT-PCR Assay

**Figure 1:**
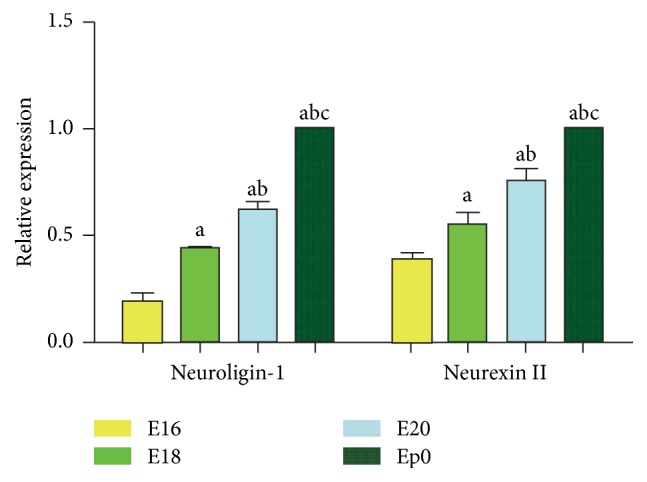
Relative expression of neuroligin-1 and neurexin II*α* mRNA on different embryonic days. Comparisons of the expressions showed that neuroligin-1 and neurexin II mRNA increased gradually along groups E16, E18, E20, and Ep0, and comparisons within groups were statistically significant (*P* values < 0.05). ^a^*P* < 0.05 versus E16, ^b^*P* < 0.05 versus E18, and ^c^*P* < 0.05 versus E20.

The qRT-PCR assay was applied for detecting the abundance of neuroligin-1 and neurexin II at mRNA level.  Table 3 and Figure 1 represent the relative expressions of groups E16, E18, E20, and Ep0. Each mRNA of neuroligin-1 and neurexin II showed a gradually increasing expression along groups E16, E18, E20, and Ep0, and comparisons within groups were statistically significant (*P* values < 0.05). This indicated a temporal trend that the expressions of neuroligin-1 and neurexin II were gradually increased during the development of ENS in the later stage of the embryo.

### 3.2. Western Blot Analysis

**Figure 2:**
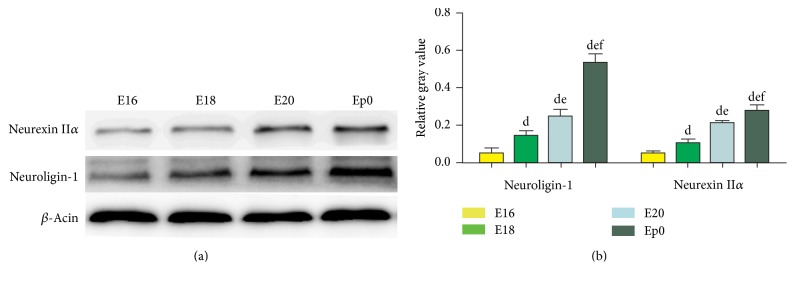
Abundance of proteins neuroligin-1 and neurexin II*α* in groups E16, E18, E20, and Ep0 by western blot. Representative blots (a) and comparisons of relative gray values (b) uncovered that proteins neuroligin-1 and neurexin II*α* showed expression from the lowest to the highest value from E16 to E18, E20, and Ep0, which further confirmed the temporal increasing trend of neuroligin-1 and neurexin II expressions. ^d^*P* < 0.05 versus E16, ^e^*P* < 0.05 versus E18, and ^f^*P* < 0.05 versus E20.

The western blot assay was performed to investigate the abundance of neuroligin-1 and neurexin II*α* proteins in the later stage of the embryo, in order to further confirm the temporal increasing trend of neuroligin-1 and neurexin II expressions. Representative blots were shown in Figure 2(a), and the relative gray values were calculated and shown in Figure 2(b) and Table 4. As is shown in Figure 2, neuroligin-1 and neurexin II*α* proteins were expressed in groups E16, E18, E20, and Ep0 with an increasing trend, and the comparisons within the groups were statistically significant (*P* values < 0.05). The trend was consistent with the finding in qRT-PCR assay.

### 3.3. Immunohistochemistry Staining

**Figure 3:**
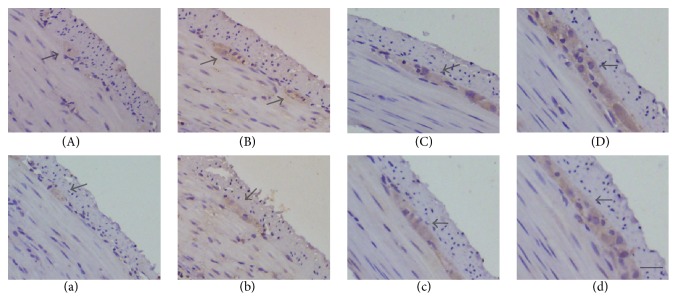
The histological appearance and expressions of neuroligin-1 and neurexin II*α* by immunohistochemistry staining (magnification ×400). The positively stained cells of both neuroligin-1 (A, B, C, and D) and neurexin II*α* (a, b, c, and d) concentrated mostly in the myenteric plexus and the expression level of neuroligin-1 and neurexin II*α* increased gradually along groups E16 (A, a), E18 (B, b), E20 (C, c), and Ep0 (D, d).

Immunohistochemical staining was employed to explore the histological appearance of proteins neuroligin-1 and neurexin II*α*. The histological appearance of neuroligin-1 (A, B, C, and D) and neurexin II*α* (a, b, c, and d) is shown in Figure 3. The positively stained cells of neuroligin-1 and neurexin II*α* were observed mostly in the myenteric plexus, being consistent with previous findings [12]. The abundance and density of positively stained cells of each specimen also indicated that expression levels of neuroligin-1 and neurexin II*α* increased gradually with the growth of the embryo. They all uncovered the temporal increasing trend of neuroligin-1 and neurexin II expressions in the later stage of the embryo.

## 4. Discussion

Proper development of enteric neurons and their circuits is vital to ensure normal intestinal function. Although certain genes such as SOX10, RET, EDNRB, ECE1, and GDNE play important roles during the time required for maturation of ENS [11, 13–15], the pathogenesis of the development of ENS still remains unclear especially in terms of formation and function of synapses. But the functional synapses are a vital foundation of a proper ENS.

It was well studied that the transsynaptic neurexin-neuroligin complex participated in the formation of functional synapses in vitro [3, 4, 16, 17] and is essential for the functional organization of synapses by mediating Ca^2+^ channels in the CNS [18]. The disruptions of neurexin and neuroligin were associated with a spectrum of cognitive disorders such as autism spectrum disorder and schizophrenia [19–21]. The patients with autism spectrum disorder or schizophrenia present a functional gastrointestinal abnormality at the same time. The numerous similarities between CNS and ENS indicated that neuroligin and neurexin also play an analogous role in ENS. This could be verified by neurexin and neuroligin expressing in postsynapse and presynapse of ENS, respectively, and their functions in the pathogenesis of HSCR, as reported in our previous study [12].

Synapses and neuron circuits are a vital foundation of functional ENS. The interaction of neurexin and neuroligin can induce both presynaptic and postsynaptic terminals differentiation and meditate synaptogenesis of both excitatory and inhibitory synapse and maintain their balance in vitro [3, 4]. The overexpression of neuroligin enhances the number of both excitatory and inhibitory synapses, and RNA interference-mediated suppression of neuroligin or blocking neuroligin-neurexin with soluble neurexins reduces the number of both excitatory and inhibitory synapses [22]. In the present study, the expression levels of neuroligin-1 and neurexin II increased gradually along the growth of the embryo, indicating a temporal correlation with the development of ENS in the later stage of the embryo. So, the increasing expressions can trigger a more functional synapse in neuron circuits and maintain the balance between excitatory and inhibitory synapses.

NIM represents a spectrum of developmental disorders of ENS. Previous studies on the pathogenesis of NIM were mainly focused on the migration and colonization of ENCDCs. The failure colonization of ENCDCs can cause HSCR [10, 11], but it cannot explain all cases of HSCR, for example, the jump type of HSCR. The abnormal ENS of some NIM infants turns to normal as the neurons continue to develop beyond birth [23] and, in clinical symptoms of many NIM infants, especially in neonates, disappear after conservative treatment, such as anal dilatation and intestinal lavage with warm normal saline. These all indicated that defects in the ENS development after the gut is colonized by ENCCs and even beyond birth also can lead to an abnormal ENS. Although the pathogenesis of NIM remains unclear, it became apparent that the anomalies of quantity, quality, and/or type of ENS impaired the motility function of the intestine [24–26].

During the development of ENS, neurons found synapses with neurons or nonneurons, such as smooth muscle cells, to form a functional network. If any factors interrupt the intrinsic increasing trend of neuroligin and neurexin, the expressions will remain at a lower level. The failure function of them will damage the number and balance of functional excitatory and inhibitory synapses and affect the function of ENS significantly as a consequence. The anomalies of ENS impaired the motility of the intestine and may lead to NIM finally. This also can explain our previous findings that neurexin and neuroligin are expressed at a significantly downregulated level in the aganglionic segment of HSCR with relative neurotransmitters, Glu and GABA, and unbalance in serum of HSCR [27, 28].

Obviously, our present conclusions only provide basic information and further investigation is needed. We hope that our conclusions provide a helpful perspective for further investigation of ENS formation and pathogenesis of NIM.

## Figures and Tables

**Table 1:** Detailed information of antibodies.

Antigen	Antibody	Dilution	Application	Source
Neuroligin-1	Mouse anti-rat monoclonal	1/650	Detect Nlgn-1 with immunohistochemistry	Abcam, USA
Neuroligin-1	Mouse anti-rat monoclonal	1/500	Detect Nlgn-1 with western blot	Abcam, USA
Neurexin II*α*	Rabbit anti-rat polyclonal	1/650	Detect Nrxn II*α* with immunohistochemistry	Abcam, USA
Neurexin II*α*	Rabbit anti-rat polyclonal	1/400	Detect Nrxn II*α* with western blot	Abcam, USA
*β*-Actin	Mouse anti-rat monoclonal	1/200	Western blot internal reference	ZSGB-BIO, China
IgG (H+L)	Horseradish peroxidase-conjugated goat anti-mouse	1/1000	Label Nlgn-1 with immunohistochemistry	ZSGB-BIO, China
IgG (H+L)	Horseradish peroxidase-conjugated goat anti-mouse	1/5000	Label Nlgn-1 with western blot	ZSGB-BIO, China
IgG (H+L)	Horseradish peroxidase-conjugated goat anti-rabbit	1/1000	Label Nrxn II with immunohistochemistry	ZSGB-BIO, China
IgG (H+L)	Horseradish peroxidase-conjugated goat anti-rabbit	1/5000	Label Nrxn II with western blot	ZSGB-BIO, China
IgG (H+L)	Horseradish peroxidase-conjugated goat anti-mouse	1/5000	Label *β*-actin with western blot	ZSGB-BIO, China

**Table 2:** Detailed information of primers.

Primers	Primer sequences (5′-3′)	Annealing temperature (°C)	Product size (bp)
Neuroligin-1	F: CGGCTTGAATTTGACTGGGAA	59	284
	R: GCAGCAAGTAGCTCCCATTG		

Neurexin II	F: TCTCCCACGAAGAGGAAACG	59.4	257
	R: GGAAATAAACGAACGGCGCA		

*β*-Actin	F: CCACCATGTACCCAGGCATT	60	243
	R: ACGCAGCTCAGTAACAGTCC		

F: upstream primer; R: downstream primer.

**Table 3:** The detailed relative expressions of neuroligin-1 and neurexin II mRNA (mean ± SD, *n* = 18).

	E16	E18	E20	Ep0
Neuroligin-1	0.189 ± 0.043	0.431 ± 0.021^a^	0.621 ± 0.036^ab^	1.000 ± 0.000^abc^
Neurexin II	0.390 ± 0.029	0.552 ± 0.054^a^	0.748 ± 0.065^ab^	1.000 ± 0.000^abc^

^a^ *P* < 0.05  *versus*  E16, ^b^*P* < 0.05  *versus*  E18, and ^c^*P* < 0.05  *versus*  E20.

**Table 4:** The detailed relative gray values of proteins neuroligin-1 and neurexin II*α* (mean ± SD, *n* = 20).

	E16	E18	E20	Ep0
Neuroligin-1	0.053 ± 0.025	0.146 ± 0.024^d^	0.249 ± 0.034^de^	0.533 ± 0.049^def^
Neurexin II*α*	0.054 ± 0.009	0.106 ± 0.020^d^	0.215 ± 0.013^de^	0.280 ± 0.030^def^

^d^ *P* < 0.05  *versus*  E16, ^e^*P* < 0.05  *versus*  E18, and ^f^*P* < 0.05  *versus*  E20.
